# Design and Analysis of an Intelligent Toilet Wheelchair Based on Planar 2DOF Parallel Mechanism with Coupling Branch Chains

**DOI:** 10.3390/s21082677

**Published:** 2021-04-10

**Authors:** Xiaohua Shi, Hao Lu, Ziming Chen

**Affiliations:** 1 School of Mechanical Engineering, Yanshan University, Qinhuangdao 066004, China; xhshi@ysu.edu.cn (X.S.); chenzm@ysu.edu.cn (Z.C.); 2 Academy for Engineering & Technology, Fudan University, Shanghai 200433, China

**Keywords:** toilet wheelchair, coupling branched chain, attitude adjustment, parallel mechanism

## Abstract

Due to the fixed size of the structure or the possibility of only simple manual adjustment, the traditional toilet wheelchair cannot easily be adapted to the size of the user or the toilet. In this paper, a planar two-degree-of-freedom parallel mechanism with coupling branch chains is proposed to enable both seat height adjustment and body posture adjustment of a toilet chair, solving the problems of posture adaptability between the user and the machine, and height matching in the process of using the wheelchair-assisted toilet. The model of the parallel mechanism was designed after analyzing the general rules of posture transformation in the human body before and after the toilet process, and the dimensions of each linkage were then determined according to the constraint conditions. By analyzing the degree of freedom, kinematics, workspace, singularity and position of the center of gravity, the rationality of the design was ensured. The weighted average function was used to find the optimal fixed point of the horizontal moving slider, and the actual trajectory at the end of the single driving mode was close to the ideal trajectory. The experimental results show that the adjustable seat height range is 290~550 mm and the adjustable angle range is 0~90°, which can enable disabled people to use the toilet independently.

## 1. Introduction

The impairment of lower extremity motor function seriously restricts the mobility of the disabled elderly and affects their quality of life [1,2]. They often face mobility problems, social discrimination, mental health problems and other problems, including daily toilet problems [3,4]. In order to reduce the burden on families and society, there is a need to solve the toilet problem for these patients, while taking into account mobility issues [5]. Without the assistance of nursing staff, going to the toilet is a difficult process for the elderly and patients with physical disabilities who cannot take care of themselves [6]. In the process of going to the toilet, it is easy for such patients to fall and risk injury. Before and after going to the toilet, there is also the problem of transferring a patient from a bed to the toilet. However, due to the need for privacy during the toilet process, the presence of nursing staff during this process will create a great psychological burden for patients [7]. Therefore, it is of social value and practical significance to develop a safe and stable wheelchair that can enable patients to complete the toilet process independently. 

In order to solve the toilet problem, scholars have conducted a considerable amount of research [8,9]. The wheelchair is the most commonly used assistive device for people with mobility difficulties, and can help patients with basic mobility and transport. Recently, with the progress of technology, intelligent wheelchairs with various auxiliary functions have been developed rapidly, and these are a research hotspot [10]. The functional designs of these wheelchairs have different emphases. One kind of wheelchair focuses on road traffic ability, and examples include the ToPChair-S [11] electric wheelchair developed in France and the Scalevo [12] electric wheelchair developed in Switzerland. These kinds of wheelchair adopt the form of a crawler driver, which can automatically adjust the posture in the process of climbing at a large inclination angle to keep the center of gravity stable. Another type of chair focuses on intelligent control for severely paralyzed people, examples including an electric wheelchair developed in South Korea that controls wheelchair movement by collecting eye movement information [13] and an electric wheelchair developed by the Iwate University of Japan that controls wheelchair movement by detecting tongue movement information [14]. Some scholars have also studied wheelchair-assisted propulsion systems, which can significantly improve road traffic capacity for wheelchairs [15,16]. In addition, some wheelchairs use artificial intelligence for autonomous navigation and multimodal interaction. The above intelligent wheelchairs were designed to meet the needs of walking without considering toilet problems. Most of the posture adjustment structures used in these wheelchairs adopt a single-degree-of-freedom vertical lifting structure, which is not flexible, and, at the same time, the weight and volume of the chair are large, resulting in more difficult transportation and handling [17,18].

As a toilet wheelchair needs to adapt to the multi-position posture changes of users in the process of going to the toilet, the design of a toilet wheelchair posture adjustment structure is the focus of many researchers [19,20]. A toilet wheelchair needs to adapt to the narrow toilet space, variable body sizes and different toilet specifications, which brings challenges to the design of a posture adjustment structure, especially considering the necessary travel function of the wheelchair. The traditional wheelchair posture adjustment structure is a simple pair of bolts, which can only achieve basic height or angle adjustment [21,22]. If a nurse has to manually rotate a handle to change the posture of the wheelchair, the user cannot control it independently; thus, they cannot use the toilet independently. Most of the structures that can automatically adjust the attitude are series link structures [23,24]. This type of structure changes the angle between the chair seat and the back of the wheelchair through the telescopic change in the length of the connecting rod in order to achieve posture adjustment. The connection mode of the series connecting rod structure is relatively simple, and the technology of the main parts is relatively mature and can meet the needs of some disabled people [25,26]. However, the series connecting rod structure is unstable and may shake in the standing state, which can be a source of fear for the user [27]. In addition, the posture transformation trajectory of the series structure is fixed, meaning that the height of the seat in the sitting position cannot be changed; therefore, it cannot adapt to the height of the user or the height of a toilet.

In view of the shortcomings of the above adjustment mechanisms, we propose a design scheme of a planar two-degree-of-freedom parallel mechanism with coupling branch chains to realize the posture adjustment function for a toilet wheelchair. Compared with the multi-link series adjustment mechanism, the parallel mechanism always forms a triangular support for the chair seat in the process of attitude adjustment, which greatly improves the stiffness and stability. The structure can be switched between a single degree of freedom and two degrees of freedom through the use of two sliders. The vertical translation of the chair seat is realized in a single degree of freedom, and the posture adjustment in the toilet process is realized in two degrees of freedom. Through the combination of seat height and tilt angle, the chair can meet the requirements of different body sizes and toilet heights. At the same time, the mechanism requires fewer components and occupies a smaller volume under the chair seat, which can leave enough space for the toilet without increasing the overall width of the wheelchair; therefore, the toilet wheelchair can easily be placed on the toilet and reduce the user’s operational difficulty.

The design scheme proposed in this paper can effectively solve the problem of patients’ independent toilet use and travel, improve the quality of life of the disabled elderly, maintain their dignity and effectively reduce the need for assistance from nursing staff. In addition, according to the information currently available, there are no other intelligent wheelchair devices using similar schemes. The main contributions of this study include the following.

1. A planar two-degree-of-freedom (2-DOF) parallel mechanism with coupling branch chains is proposed. The structure is compact, which not only reserves enough space for the toilet seat, but also enables adjustment of the height and posture of the chair seat at the same time so that disabled people can go to the toilet independently.
2. By analyzing the constraint conditions in the operation process, the actual dimensions of the connecting rod of the parallel mechanism are determined, and the model design and the test platform are completed.
3. The coupling relationship between seat height and seat inclination adjustment ability is analyzed, and attitude adjustment trajectory planning is completed.
4. The weighted average function is used to find the optimal fixed point of the horizontal moving slider, and the actual trajectory of the end is close to the ideal trajectory.

## 2. Design of Attitude Adjustment Parallel Mechanism

### 2.1. Wheelchair Size Design Based on Ergonomics

**Figure 1:**
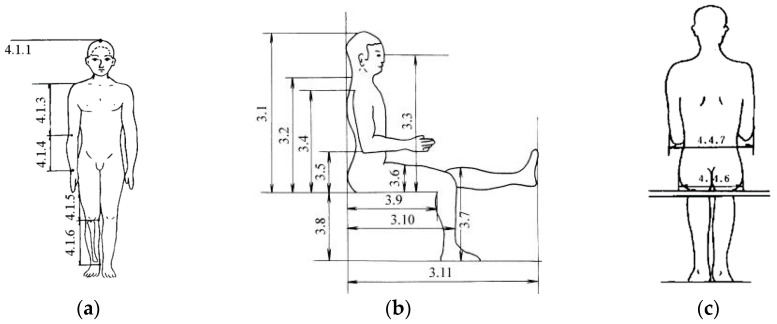
Chinese adult human body size: (**a**) standing size; (**b**) side-view size in sitting position; (**c**) back-view size in sitting position.

The design of the toilet wheelchair needs to consider the distribution of human body sizes. The main parameters of the wheelchair were designed in accordance with the data on the national standard of human body size for adults in China [28], as shown in Figure 1 and Table 1.

**Table 1:** Chinese adult body size.

Measurement Items (mm)	Percentage of Male (18–60 Years)					Percentage of Female (18–60 Years)				
	5	10	50	90	95	5	10	50	90	95
3.1 sitting height	858	870	908	947	958	809	819	855	891	901
3.4 shoulder height	557	566	598	631	641	518	526	556	585	594
3.7 knee height	456	464	493	523	532	424	431	458	485	493
3.8 calf height	383	389	413	439	448	342	350	382	399	405
3.9 seat depth	421	429	457	486	494	401	408	433	461	469
3.10 hip-to-knee distance	515	524	554	585	595	495	502	529	561	570
4.4.6 hip breadth	282	288	306	327	334	290	296	317	340	346
4.4.7 elbow width	371	381	422	473	489	348	360	404	460	478

According to the human body size data, the final basic size parameters of the wheelchair are shown in Table 2.

**Table 2:** Basic parameters of toilet wheelchair.

Wheelchair Main Parameters	Parameter Value
Seat width (mm)	460
Seat depth (mm)	440
back height (mm)	550
Angle between back and seat in sitting position (°)	105
Angle between leg support and seat (°)	105

### 2.2. Analysis of Toilet Process

**Figure 2:**
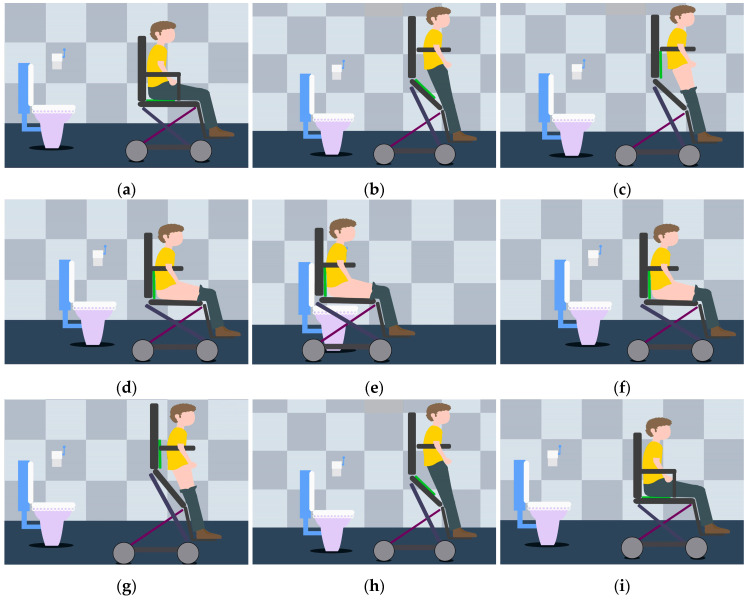
The process of using a wheelchair to assist with toilet use: (**a**) wheelchair into toilet; (**b**) sitting position changes to standing position; (**c**) preparation before going to the toilet; (**d**) from standing to sitting; (**e**) wheelchair sits astride the toilet; (**f**) wheelchair away from toilet; (**g**) sitting position changes to standing position; (**h**) fix clothes after using the toilet; (**i**) wheelchair pulled out of the bathroom.

When using a wall-mounted toilet, the human body leans forward, and it is easy to lose stability when using a wheelchair. Therefore, this study mainly focuses on sitting toilets. The premise was to design the posture adjustment mechanism by dividing the toilet process into stages and understanding the posture transformation laws at each stage. The process of using a wheelchair to assist toilet use is shown in Figure 2.

The whole toilet process can be summarized as a reciprocating posture adjustment process from a standing posture to a sitting posture and then a standing posture again. The armrest of the toilet wheelchair passes under the armpit of the user, and a bandage is fixed from the knee to the leg to protect the user. The chair seat lifts the patient’s body upward to realize the adjustment from a sitting posture to a standing posture. Clothing is removed and organized in the standing posture, while toilet use and cleaning are completed in the sitting posture. When using the toilet, the lower end of the chair seat should be attached to the upper end of the toilet. However, according to the national standard of sanitary ceramics [29], each toilet has different specifications in height and width, and special toilets designed for the elderly and the disabled have a higher height. Therefore, a toilet wheelchair should be adaptable to a good range of toilet heights and widths. In addition, considering the narrow toilet space in family and nursing homes, the transportation process before and after toilet use may lead to collision with obstacles, thus causing injury to patients. Based on the comprehensive analysis of the toilet process and the transportation before and after toilet use, the following requirements were put forward for the design of the posture adjustment mechanism of our toilet wheelchair: the height of the chair seat should be adjustable; a large range of posture adjustments should be realizable between the chair seat and back; it should be easy to arrange the omni-directional drive train and there should be safety protection measures for the posture transformation process.

### 2.3. Structure Diagram of Parallel Mechanism for Attitude Adjustment

**Figure 3:**
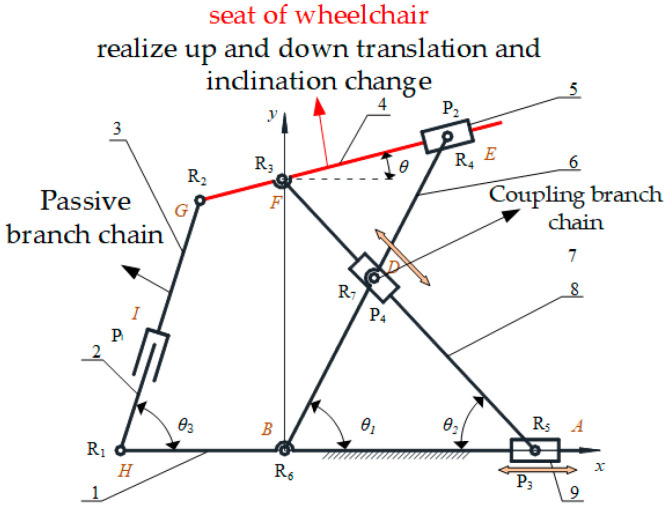
A planar two-degree-of-freedom (2-DOF) parallel mechanism with coupling branches: 1. link 1; 2. link 2; 3. link 3; 4. link 4; 5. slider 2; 6. link 5; 7. slider 3; 8. link 6; 9. slider 1. *R*_1_, *R*_2_, *R*_3_, *R*_4_, *R*_5_, *R*_6_ and *R*_7_ represent the revolute joints. *P*_1_, *P*_2_, *P*_3_ and *P*_4_ represent the prismatic pairs. *θ*, *θ*_1_, *θ*_2_ and *θ*_3_ represent the angles between link 4, link 5, link 6, link 2 and the horizontal plane, respectively.

As the patient sits on the chair seat, the patient’s posture adjustment is mainly realized by the change in chair surface posture. A toilet wheelchair has both height and angle adjustment functions; therefore, the seat adjustment mechanism should have at least two degrees of freedom. Based on the research on sitting up during the toilet process in the previous section, we propose a planar 2-DOF parallel mechanism with coupling branch chains to realize attitude adjustment, as shown in Figure 3.

The mechanism shown in Figure 3 is a unilateral support structure under the chair seat. The left and right supporting mechanisms under the chair seat are identical; thus, only one side was selected for analysis. As the seat is fixedly connected to connecting link 4, the relationship between the height and angle of the seat can be simplified to the attitude change of connecting link 4. In this mechanism, the prismatic pair *P*_4_ and the revolute joint *R*_7_ are coupled to form a coupling branch chain. The passive rotary–prismatic–rotary (RPR) branch chain is formed with connecting link 3, connecting link 2 and prismatic pair *P*_1_, and the RPR branch chains on both sides are connected with a pedal for patients to step on. The lengths of connecting links 6 and link 7 are equal; driving horizontal moving pair *P*_3_ can adjust the height of the chair seat and jointly driving the moving pair *P*_4_ at the cross node and the lower horizontal moving pair *P*_3_ can adjust the wheelchair posture.

### 2.4. Analysis of Degree of Freedom

The existence of coupling branch chains increases the difficulty of mechanism analysis. In the preliminary analysis, the coupling of the parallel mechanism is removed to form a planar RPR–RRP–PRR parallel mechanism. After solving the degree of freedom of the simplified parallel mechanism, coupling branch chains are added to solve the degree of freedom of the parallel mechanism with coupling branch chains. 

According to the derivation of reference [30], the constrained screw system of the mechanism in decoupling state is determined as shown in Equation (1).

(1) $$\{\begin{array}{c} {\$}_{1}^{r}=(\begin{array}{ccccccc} 0 & 0 & 0 & ; & 1 & 0 & 0 \end{array})\\ {\$}_{2}^{r}=(\begin{array}{ccccccc} 0 & 0 & 0 & ; & 0 & 1 & 0 \end{array})\\ {\$}_{3}^{r}=(\begin{array}{ccccccc} 0 & 0 & 1 & ; & 0 & 0 & 0 \end{array}) \end{array}$$

According to the Kutzbach–Gübler degree of freedom formula, the degree of freedom *M* = 3 in a decoupling state can be calculated. The coordinate system *Bxyz* shown in Figure 2 is established. When there are coupling branches, the constraint screw of the three branches of the parallel mechanism is obtained as follows:

(2) $$\{\begin{array}{c} {\$}_{4}^{r}=(\begin{array}{ccccccc} 0 & 0 & 0 & ; & 1 & 0 & 0 \end{array})\\ {\$}_{5}^{r}=(\begin{array}{ccccccc} 0 & 0 & 0 & ; & 0 & 1 & 0 \end{array})\\ {\$}_{6}^{r}=(\begin{array}{ccccccc} 0 & 0 & 1 & ; & 0 & 0 & 0 \end{array}) \end{array}$$

where ${\$}_{4}^{r}$, ${\$}_{5}^{r}$ and ${\$}_{6}^{r}$ represent the constraint helix of the first, second and third branches of the parallel mechanism with coupling branch chains, respectively. It can be seen from Equation (2) that the two mutually perpendicular constraint couples and the binding force of the spiral system are still the three common constraints of the planar mechanism, as in the decoupled state. Therefore, the order of the mechanism does not change due to the generation of coupling branched chains, but rather only increases the number of components, the number of motion pairs and the number of redundant constraints in the mechanism; thus, *M* = 2 can be obtained. Namely, the mechanism has two degrees of freedom with coupling support chains. Therefore, taking any two motion pairs of the mechanism as the driving input objects can make the mechanism realize a certain motion trajectory. According to the design principle that the space under the seat is reserved for the toilet as much as possible, and considering the rationality of the structure design, the drive layout scheme was screened, and finally, two prismatic pairs, *P*_3_ and *P*_4_, were selected as the drive input.

## 3. Connecting Rod Size Determination and Structure Design

### 3.1. Constraint Index of Rod Length

In the parallel mechanism described in the previous section, the length of the *L*_6_ and *L*_8_ links determines the overall structure size and attitude adjustment range of the wheelchair, which were the first parameters to be determined before design, and the optimal length of these two links needed to be determined according to multiple index requirements [31]. For the convenience of study, let *AB* = *L*_1_, *AD* = *L*_2_, *BE* = *L*_6_ and *AF* = *L*_8_. According to the basic indexes in Table 2 and the body size distribution of the adult population of China, the initial range of *BE* and *AF* can be determined as follows:

(3) $$300\;\leq \;L_{6}\;\leq \;800\;\mathrm{mm},\;300\leq L_{8}\leq 800\;\mathrm{mm}$$

In view of the fact that the wheelchair cannot have an abnormal posture that causes discomfort to the patient in the manned state [32], and in order to reduce the search range of the optimization index, the position of the hinge point *D* was appropriately limited:

(4) $$\begin{array}{l} (L_{6}+L_{8})/5\leq L_{1}\leq 5(L_{6}+L_{8})/12\\ 2L_{8}/5\leq L_{2}\leq 7L_{8}/8 \end{array}$$

Formula (4) was the basic constraint based on empirical value. The maximum reachable angle *θ*_max_, minimum reachable angle *θ*_min_, maximum reachable height *H*_max_ and minimum reachable height *H*_min_, which are most closely related to the seat height and attitude adjustment, were selected as evaluation indexes to establish a quantifiable evaluation system to determine the connecting rod size.

By setting the coordinates of point *E*(*E_x_*, *E_y_*) and point *F*(*F_x_*, *F_y_*), the inclination angle of the moving platform *θ* can be expressed as:

(5) $$\theta =\{\begin{array}{ll} \arctan(\Delta y/\Delta x) & \left(E_{x}>F_{x}\right)\\ 90{}^{\circ} & \left(E_{x}=F_{x}\right)\\ \arctan(\Delta y)/\Delta x)+180{}^{\circ} & \left(E_{x}<F_{x}\right) \end{array}$$

where Δ*x* = *F*_x_ − *E*_x_, Δ*y* = *F*_y_ − *E*_y_. According to Helen’s theorem, the height of the moving platform *H* can be expressed as:

(6) $$H=4S_{\Delta }/L_{1}$$

where $S_{\Delta }=\sqrt{p(p-BD)(p-AD)(p-AB)}$ and p=(BD+AD+AB)/2. In addition, the proportional relationship between rod *BE* and rod *AF* needs to be limited; thus, $L_{6}/2\leq L_{8}\leq 2L_{6}$. The boundary conditions of the evaluation indexes can be calculated as shown in Table 3, where $\xi =L_{6}+L_{8}$.

**Figure 4:**
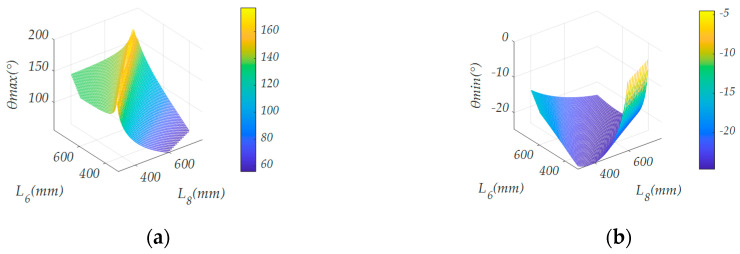

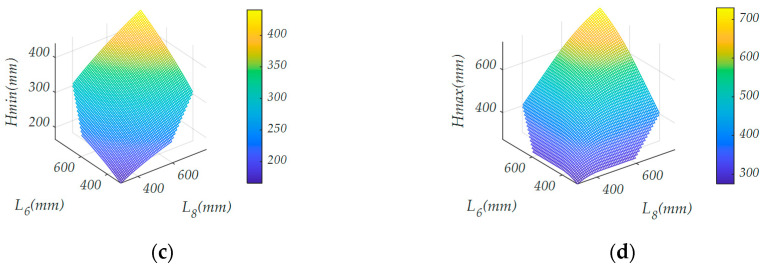
The relationship between each evaluation index and the length of two connecting rods: (**a**) the relationship between the maximum reachable angle and the length of the connecting rod; (**b**) the relationship between the minimum reachable angle and the length of the connecting rod; (**c**) the relationship between the maximum reachable height and the length of the connecting rod; (**d**) the relationship between the minimum reachable height and the length of the connecting rod.

The relationship between each evaluation index and the length of two connecting rods is shown in Figure 4.

According to the actual situation, the seat (the fourth connecting rod) needs to adapt to the size of the toilet. In addition, there is usually a 20 ± 10 mm pad above the toilet. Considering the tire size of the wheelchair, *H*_max_ ≥ 300 mm and *H*_min_ ≤ 290 mm are limited. The inclination angle of the seat should exceed the range between the horizontal sitting position and the vertical standing position so that −20° ≤ *θ* ≤ 120°. As a greater height adjustment ability not only facilitates the use of different specifications of toilets for patients but also expands the ability of patients to pick up items in daily life, *ΔH* ≥ 160 mm is limited, where *ΔH = H*_max_ − *H*_min_. To meet the design requirements, the smaller the sum of *L*_6_ and *L*_8_ is, the more conducive it is to reducing the overall size of the wheelchair. In order to avoid instability of the center of gravity caused by the large deviation of the seat during the lifting process, *L*_6_ and *L*_8_ should be as close in value as possible. According to the above requirements, the range and boundary conditions of the rod length can be determined. In order to further obtain the optimal solution of the length of the connecting rod, the above factors were quantified and the following evaluation function was established:

(7) $$Z=\sum_{i=1}^{4}\mu_{i}k_{i}\;k_{1}=(\theta_{\max}-\theta_{\min}),\;k_{2}=(H_{\max}-H_{\min})/(H_{\max}+H_{\min}),\;k_{3}=-(L_{6}+L_{8})/500,\;k_{4}=-\left|L_{6}-L_{8}\right|$$

where *μ_i_*(*i* = 1,2,3,4) represents the weighting factor, taking 0.2, 0.2, 0.3 and 0.3, respectively; *k*_1_ represents the angle adjustment ability factor of the moving platform; *k*_2_ represents the height adjustment ability factor of the moving platform; *k*_3_ represents the length factor of the connecting rod; and *k*_4_ represents the stability factor. The optimal bar length was selected through the evaluation function score. At the maximum value of the evaluation function, the optimal solution of the length was obtained, and the length *L*_6_ = 440 mm and *L*_8_ = 440 mm. 

Considering the universality of wheelchair seat size [33], *GF* = 50 mm was determined. In order to ensure the user’s comfort in the process of sitting, standing and state transition between the two postures, the inclination angle of the passive branch chain should conform to the laws of human movement, and *BH* = 145 mm was selected. After the length of the link was determined, *HI* = *GI* = 220 mm was selected to ensure that the second link and the third link cooperate stably and do not interfere in the process of wheelchair posture transformation from a sitting posture to a standing posture. Through the above analysis, the lengths of the main connecting rods of the parallel mechanism were preliminarily determined.

### 3.2. Workspace and Singularity Analysis

In order to ensure the safety of patients, singularity must be avoided in the process of toilet wheelchair posture adjustment. According to the forward and inverse kinematics of the parallel mechanism [34], it is clear that the movement ranges of *P*_3_ and *P*_4_ are *L*_1_ = 170~360 mm and *L*_2_ = 220~375 mm, respectively. When the *P*_3_ and *P*_4_ moving pairs move within the above range of motion, the inclination of the moving platform will be greater than 90°. However, because the chair seat inclination cannot be too large or too small in the actual use of the wheelchair, the chair seat inclination also becomes a limitation of the working space. In addition, the lengths of the upper and lower connecting rods of the passive branch chain have been determined. Considering that the radius of the connecting rod should not be too small, the distance between *R*_1_ and *R*_2_ of the rotating pair should be limited to avoid interference between the passive branch chain of the mechanism and the moving and fixed platforms. Therefore, the following conditions should be met in the attitude adjustment process of the moving platform:

(8) $$\{\begin{array}{l} 0{}^{\circ}\leq \theta \leq 90{}^{\circ}\\ \theta_{3}\leq 90{}^{\circ}\\ \left|GH\right|\geq 230\mathrm{mm} \end{array}$$

**Figure 5:**
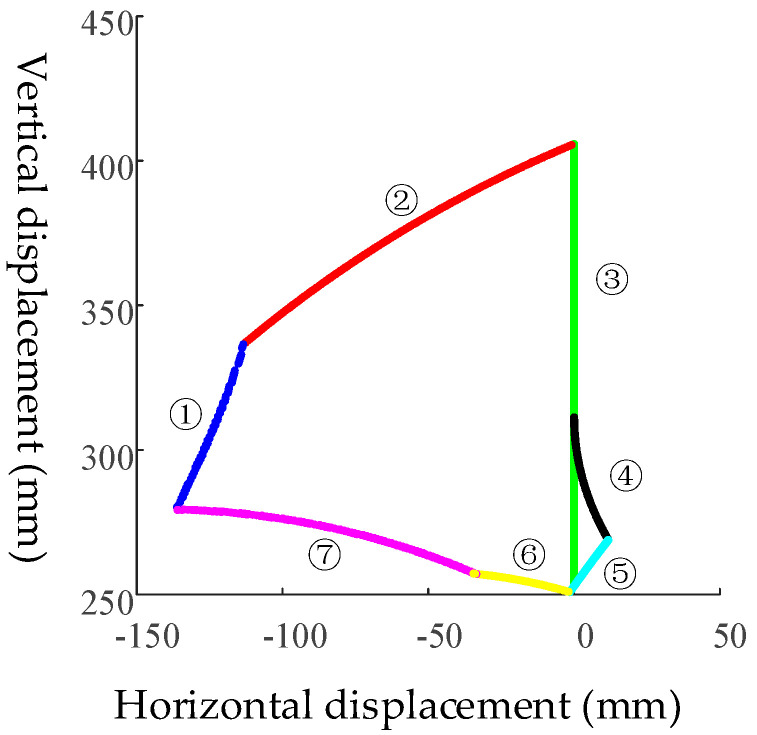
Trajectory boundary of point *F* at the end of the mechanism.

Taking point *F* as the end-point of the moving platform, the relationship between its trajectory and seat inclination is shown in Figure 5 under limited conditions.

As can be seen from Figure 5, the boundary of point *F* can be divided into seven segments:

(1) Boundary 1 is obtained when *θ*_3_ = 90°;

(2) Boundary 2 is obtained when *L*_1_ = *L*_1min_ = 170 mm;

(3) Boundary 3 is obtained when *L*_2_ = *L*_2min_ = 220 mm;

(4) Boundary 4 is obtained when *θ*_1_ + *θ*_2_ = 90°;

(5) Boundary 5 is obtained when *L*_1_ = *L*_1max_ = 360 mm;

(6) Boundary 6 is obtained when *L*_2_ = *L*_2max_ = 375 mm;

(7) Boundary 7 is obtained when *GH* = 230 mm.

In Figure 5, the upper-left region of *F* workspace boundary 4 corresponds to the situation of *θ*_1_ + *θ*_2_ > 90°, and the lower-right region corresponds to the situation of *θ*_1_ + *θ*_2_ < 90°. 

In addition, in order to avoid the problems of self-locking and excessive driving torque, it is necessary to consider the singularity of the parallel mechanism in the workspace, including the singularity of the positive solution and the singularity of the inverse solution. In order to facilitate the subsequent analysis, the input of the mechanism was converted from the sliding of the slider to the change in the inclination angle of *θ*_1_ and *θ*_2_.

The singularity analysis of positive solutions was performed first. According to the kinematics, the Jacobian matrix ***J***_θ_ of the positive solution of the parallel mechanism is obtained:

(9) $$J_{\theta }=\left[\begin{array}{cc} 220(ct_{2}c_{1}-s_{1}) & 440s_{2}-\frac{220s_{1}}{{\left(s_{2}\right)}^{2}}\\ 0 & 440c_{2}\\ A & B \end{array}\right]$$

where *ct*_2_ is cos*θ*_2_, *c*_1_ is cos*θ*_1_, *s*_1_ is sin*θ*_1_, *s*_2_ is sin*θ*_2_, *c*_2_ is cos*θ*_2_ and *A* and *B* are represented by Equation (10):

(10) $$\{\begin{array}{l} A=\frac{s_{21}+2c_{1}s_{dq}{}^{2}+(2s_{2}s_{1}-2s_{2}{}^{2})c_{21}}{(1+(\frac{2s_{2}(s_{1}-s_{2})}{s_{21}+s_{dq}}{)}^{2})(s_{21}+s_{dq}{)}^{2}}\\ B=\frac{(2c_{2}s_{1}-s_{dq})(s_{21}+s_{dq})}{(1+(\frac{2s_{2}(s_{1}-s_{2})}{s_{21}+s_{dq}}{)}^{2})(s_{21}+s_{dq}{)}^{2}}-\frac{(2s_{2}s_{1}-2s_{2}{}^{2})(s_{21}+s_{dq})c_{21}+2c_{dq}}{(1+(\frac{2s_{2}(s_{1}-s_{2})}{s_{21}+s_{dq}}{)}^{2})(s_{21}+s_{dq}{)}^{2}} \end{array}$$

where *s*_21_ denotes sin(*θ*_2_ − *θ*_1_), *c*_21_ denotes cos(*θ*_2_ − *θ*_1_) and *dq* denotes 2*θ*_2_.

When rank(***J***_θ_) < 2—that is, when all of the rows in the Jacobian are linearly independent—the singularity of the positive solution of the mechanism appears. In order to solve the singular configuration, it is assumed that the first and second lines of the Jacobian matrix are linearly independent, and then, *θ*_1_ + *θ*_2_ = π/2 or *θ*_2_ = π/2 can be obtained. When *θ*_2_ = π/2, the moving platform is in the vertical state, which is not in the value range; thus, it is omitted. Then, by substituting *θ*_1_ + θ_2_ = π/2 into the Jacobian matrix, the rank (***J****_θ_*) = 2 is obtained, which shows that there is a linear correlation between the lines of the Jacobian matrix; thus, the mechanism will not have positive solution singularity in the working range.

In addition, considering the singularity of the inverse solution, the inverse Jacobian matrix ***J***_F_ is obtained:

(11) $$J_{F}=\left[\begin{array}{ccc} -sin\theta & cos\theta & 1-\frac{Xcos\theta +Ysin\theta }{C}\\ 0 & -\frac{1}{D} & 0 \end{array}\right]\;C=440\sqrt{1-{\left(\frac{X\sin\theta -Y\cos\theta }{440}\right)}^{2}},\;D=440\sqrt{1-{\left(\frac{Y}{440}\right)}^{2}}$$

where (*X*, *Y*, *θ*) represent the pose of the moving platform.

Similarly, assuming that the first and second columns of ***J****_F_* are linearly related, we obtain the case where the singularity configuration of the inverse solution of the mechanism is *θ* = 0; that is, *X* = 0 or *θ*_1_ = *θ*_2_. When it is substituted into the Jacobian matrix of Equation 12, the second and third columns are still linearly independent. Only when *θ*_1_ = *θ*_2_ = 0—that is, when the moving platform coincides with the fixed platform—is it linearly related, but the solution is not in the range of value; thus, there is no inverse solution singular configuration in the working range.

### 3.3. The Stability of the Wheelchair in Attitude Adjustment

**Figure 6:**
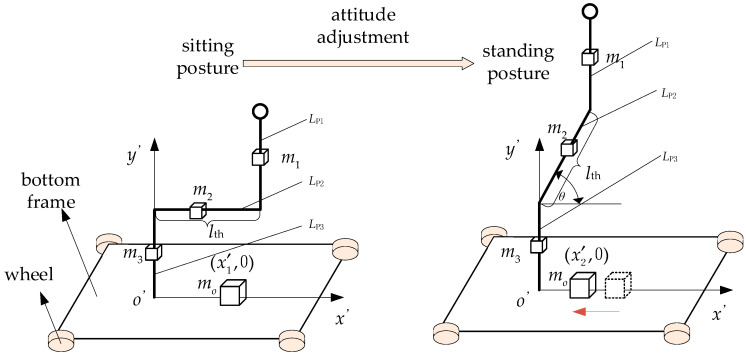
Stability analysis in attitude adjustment.

The toilet wheelchair moves slowly in the process of posture adjustment, which can be analyzed by statics [34]. The upper body, thigh and calf of the human body are, respectively, fused with the back, seat and pedal of the wheelchair to simplify them into three homogeneous connecting rods, which are, respectively, represented by *L*_p1_, *L*_p2_ and *L*_p3_, as shown in Figure 6. The center of gravity of each link is located at the mid-point of the connecting rod, and the mass of each part is expressed by m_1_, m_2_ and m_3_, respectively. The length of link 2 is expressed as *l*_th_. Link 2 is horizontal in the sitting position. Link 1 and link 3 remain upright during attitude adjustment. The coordinate system *o*’-*x*’*y*’ was established as shown in Figure 6. *x’*_1_ and *x’*_2_ represent the position of the combined body weight center on the x’-axis in the processes of sitting and posture adjustment, respectively. In the sitting position, *x’*_1_ = (*m*_1 ·_ *l*_th_ + 0.5*m*_2 ·_ *l*_th_)/(*m*_1_ + *m*_2_ + *m*_3_); during posture adjustment, the center of gravity changes and moves along the *x*’-axis, *x’*_2_ = (*m*_1 ·_ *l*_th_cos*θ* + *m*_2 ·_ 0.5*l*_th_cos*θ*)/(*m*_1_ + *m*_2_ + *m*_3_). However, in the whole adjustment process, the center of gravity will fall within the four supporting points of the frame; therefore, the toilet wheelchair and the user can remain stable.

### 3.4. Structural Design and Key Performance Analysis

This section describes the transformation of the theoretical model into a concrete physical model. In order to fully ensure the safety of users when they choose the function in the toilet environment, a mechanical limit was arranged at each movable joint of the wheelchair, and flexible safety auxiliary devices such as a small leg protection belt were added. Considering the actual needs of the elderly, the use of the controller was simplified, and the function of “one button to achieve” was set; that is, the attitude conversion can be completed automatically by pressing the button. In addition, an emergency stop switch was provided to prevent an emergency. The cables for all the electrical components should be built in as much as possible.

**Figure 7:**
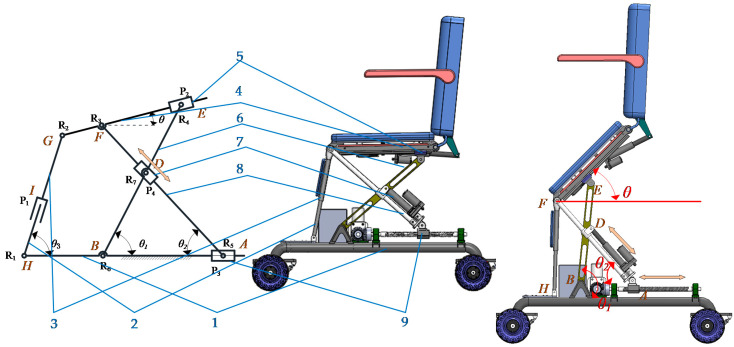
Transformation from theoretical model to physical model.

**Figure 8:**
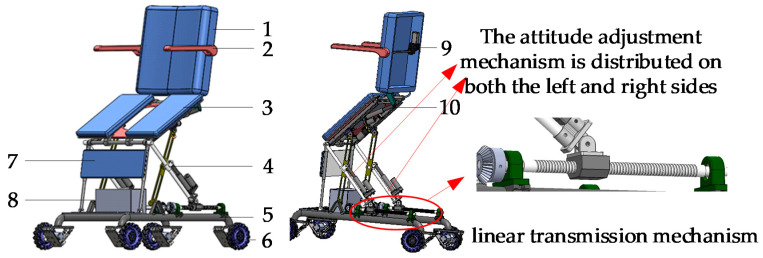
The overall structure of the toilet wheelchair: 1. back; 2. armrest; 3. seat; 4. posture adjusting mechanism; 5. chassis frame; 6. driving wheel set; 7. leg guard; 8. control box; 9. armrest adjusting motor; 10. backrest adjusting motor.

The toilet wheelchair includes three parts: an attitude adjustment mechanism, a carrying platform and a chassis. The input parts of the attitude adjustment device, *P*_3_ and *P*_4_ movable sliders, were realized using a ball-screw transmission mechanism and an electric push rod. A gyroscope was arranged below the seat for attitude feedback. The transformation from the theoretical model to the design model is shown in Figure 7. The overall structure of the toilet wheelchair is shown in Figure 8.

### 3.5. Finite Element Analysis of Key Components

The linear transmission mechanism is the key component of the toilet wheelchair. To obtain higher accuracy of the wheelchair posture adjustment process, the deformation of the lead screw must be in a reasonable range. In order to ensure the stable fit of the bevel gear, the connecting end of the lead screw and the bevel gear was fixed, while the other side was fixed or supported. Through the finite element method based on the ANSYS Workbench, the combined model of the screw and nut seat was analyzed in order to select a reasonable support mode.

**Figure 9:**
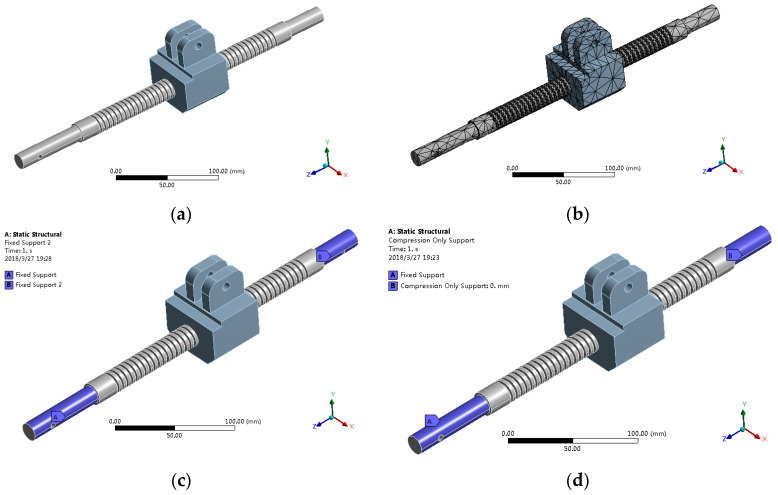
Finite element analysis pre-treatment: (**a**) model simplification; (**b**) mesh division; (**c**) two ends fixed; (**d**) one end fixed and the other end supported.

The ball-screw pair was simplified as a trapezoidal screw pair, the nut seat was simplified as a part, and then, the simplified model was imported into ANSYS, as shown in Figure 9a (corresponding position *L*_1_ = 280 mm). The material of the lead screw and nut seat was structural steel, their elastic modulus was E = 2.07 × 105 N/mm^2^ and the Poisson ratio was *μ* = 0.3. The simplified model was meshed based on the automatic meshing method, and the meshing results shown in Figure 9b were obtained. The left side of the lead screw adopts a “fixed” support mode, and the right side adopts a “fixed” support mode, as shown in Figure 9c, and a “support” mode, as shown in Figure 9d.

According to the force analysis, in the attitude adjustment process, the maximum horizontal and vertical loads on the nut seat are obtained in the sitting position, and the specific loads are shown in Table 4.

**Figure 10:**
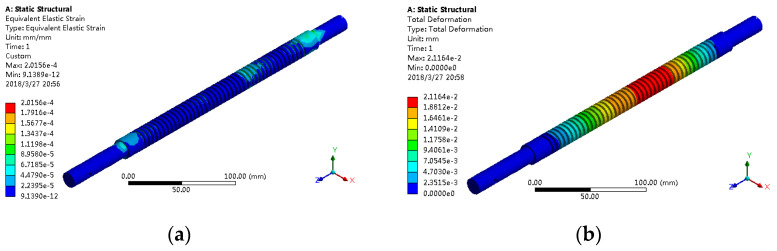
Finite element analysis results of fixed support at both ends: (**a**) equivalent elastic strain and (**b**) total deformation.

**Figure 11:**
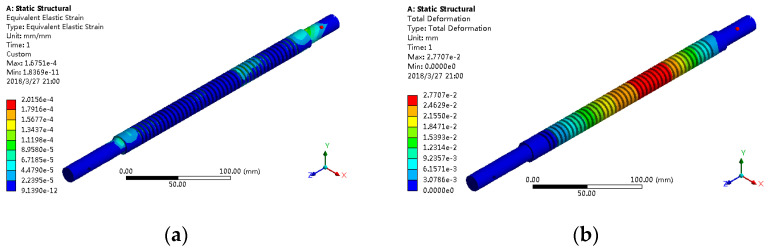
Finite element analysis results of one end fixed and one end supported: (**a**) equivalent elastic strain and (**b**) total deformation.

The finite element analysis results of two different support modes are shown in Figure 10 and Figure 11, where the maximum load was applied to the nut seat.

For both cases, the equivalent elastic strain and total deformation of the lead screw are shown in Table 5.

It was found that the equivalent elastic strain and the total deformation are small, and the results of the two are not different; under the fixed support mode at both ends, the total deformation is small. Therefore, in order to realize the linear transmission function better, the fixed support form was adopted at both ends of the screw.

## 4. Analysis of Attitude Adjustment

### 4.1. Analysis of Seat Inclination Adjustment Ability at Different Heights

In addition to meeting the requirement for patients’ autonomous toilet use, the intelligent toilet wheelchair also has the ability to assist patients in walking. Patients who need assistance when using a toilet are usually in poor physical condition and are not able to autonomously drive a wheelchair on complex roads. Therefore, the toilet wheelchair pays more attention to resolving the short-distance travel problem of patients in the community environment. 

**Figure 12:**
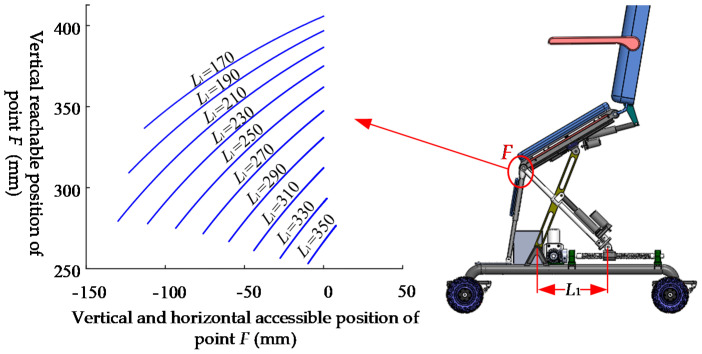
*F*-point trajectory of moving platform with different seat heights.

As a walking tool, the toilet wheelchair needs to have a certain seat tilt adjustment ability to stabilize the patient’s center of gravity, reduce discomfort and fear when moving on a sloping road and enhance the user’s safety and comfort. According to different seat heights, the effect of seat inclination adjustment will be different to some extent. The inclination change can be represented by the trajectory of point *F* on the moving platform, as shown in Figure 12.

It can be seen from Figure 11 that with the decrease in height of the chair seat, the adjustment ability of the wheelchair inclination angle decreases continuously. This is because the lower the height of the seat is, the more seriously the adjustment ability of the seat inclination is limited by the length of the bottom slide. When we travel outdoors, uneven road surfaces are often unavoidable. In order to ensure that the seat has a certain inclination adjustment ability, we should appropriately raise the seat height when encountering larger slopes or more serious potholes. When the road surface is flat, we should appropriately reduce the body height to ensure a comfortable sitting posture.

### 4.2. Seat Posture Adjustment Planning

When patients use a wheelchair for a long time, they need to adjust their posture in addition to going to the toilet. In order to extend the joints or expand the ability to pick up objects, the attitude change does not need height adjustment, but rather directly enters the angle change stage. As the parallel mechanism has two inputs, in order to reduce the complexity of the control, this section uses the single drive method to complete the attitude change and makes the actual trajectory of point *F* at the end of the moving platform close to the ideal trajectory of the two drives.

In the process of posture change, in order to ensure that the squeezing force between the lower limbs and the leg guard belt will not be too large, the inclination angle of the passive branch chain should be constrained to limit the overall center of gravity of the wheelchair from moving forward too much. Referring to the attitude change process of the existing auxiliary standing wheelchair, this wheelchair keeps the inclination angle between the passive chain and the fixed platform stable and selects *θ*_3_ = 75°. Then, the hinge point of the passive branch chain and the moving platform moves on the straight line *Y*_G_ = tan75° (*X*_G_ + *l*_BH_), where (*X*_G_, *Y*_G_) are the coordinates of point *G* in Figure 2 and *l*_BH_ is the length of the connecting rod *BH*. The trajectory formed by point *F* in this state is regarded as the ideal trajectory.

**Figure 13:**
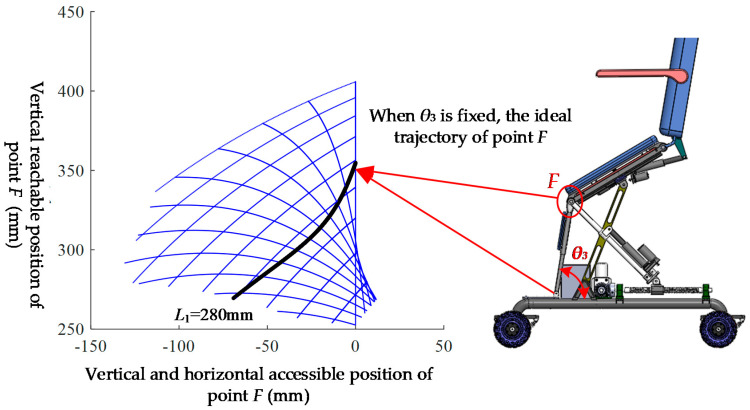
Ideal trajectory of point *F.*

The parallel mechanism has two degrees of freedom, and if one degree of freedom is arbitrarily limited, the mechanism can be changed into a single-degree-of-freedom mechanism. In order to determine the appropriate degree-of-freedom limitation scheme, the ideal trajectory is displayed on the grid workspace drawn using the univariate method, as shown in Figure 13.

**Figure 14:**
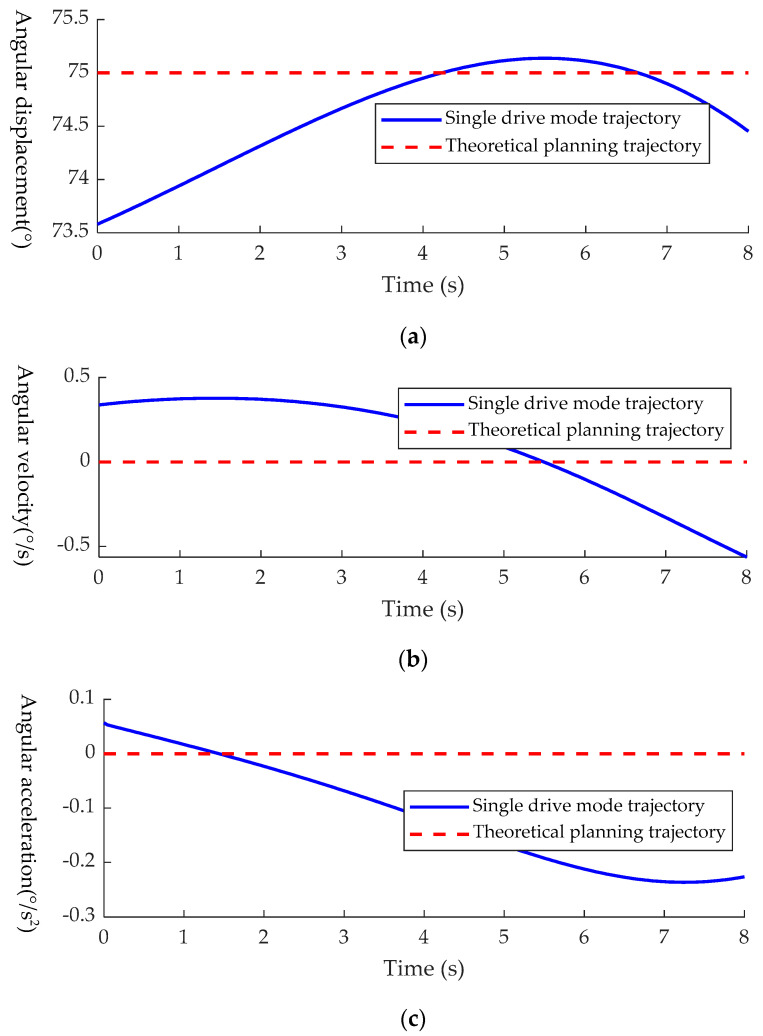
Comparison of kinematic performance between ideal mode and single driving mode: (**a**) comparison diagram of angular displacement; (**b**) comparison chart of angular velocity; (**c**) comparison diagram of angular acceleration.

The black line in Figure 12 is the ideal trajectory of point *F*. According to the observation, the ideal trajectory is close to that of *L*_1_ = 280 mm; thus, slider *P*_3_ should be locked and slider *P*_4_ should be driven in the attitude adjustment. In order to determine the best locking position of slider *P*_3_, the weighted average method was used for analysis. Considering that the end pose is more important, the closer it is to the end of the pose, the greater the set weight should be. At the same time, there should be no excessive fluctuations in the process of attitude adjustment, and the weight should be appropriately reduced in the initial stage of adjustment. In conclusion, the discrete weighted sum function is established:

(12) $${\bar{L}}_{1}=\frac{\sum_{i=1}^{N}\theta_{i}L_{1i}}{\sum_{i=1}^{N}\theta_{i}}$$

where ${\bar{L}}_{1}$ is the fixed position of *P*_3_ to be determined, *L*_1i_ is the slider position corresponding to the ith data point, *θ_i_*_0_ is the moving platform inclination corresponding to the ith data point and *N* is the total number of data points. After taking *N* = 1000 for discrete decomposition and calculation, *L*_1_ = 284.7318 mm is obtained, and *L*_1_ = 285 mm is taken for convenience of design. In order to verify the rationality of the above analysis method, firstly, the slider *P*_3_ was fixed at *L*_1_ = 285 mm; then, the slider *P*_4_ was moved separately at a speed of 15 mm/s for attitude adjustment, and the driving time was 8 s. The simulation curves of angular displacement, velocity and acceleration of point *F* can be obtained by comparing the actual trajectory of point *F* with the ideal trajectory, as shown in Figure 14.

It can be seen from the figure that in the starting position, the maximum deviation between the inclination angle of the passive branch chain, the fixed platform and the theoretical trajectory is Δ*θ*_3_ = 1.42° in the single driving mode.

It can be seen from Figure 14 that at the end point, the deviation between the inclination angle of the passive branch chain and the angular velocity and angular acceleration in the ideal trajectory reaches the maximum, in which the angular velocity deviation is $\Delta {\dot{\theta }}_{3}\;=\;0.56{}^{\circ}/s$ and the angular acceleration deviation is $\Delta {\ddot{\theta }}_{3}\;=\;0.24{}^{\circ}/s^{2}$. The actual trajectory and the ideal trajectory in the single driving mode are very small, which will not cause discomfort to patients. The rationality of the single driving mode and the fixed position of the slider *P*_3_ is therefore verified.

## 5. Experimental Verification

### 5.1. Introduction of Physical Prototype

**Figure 15:**
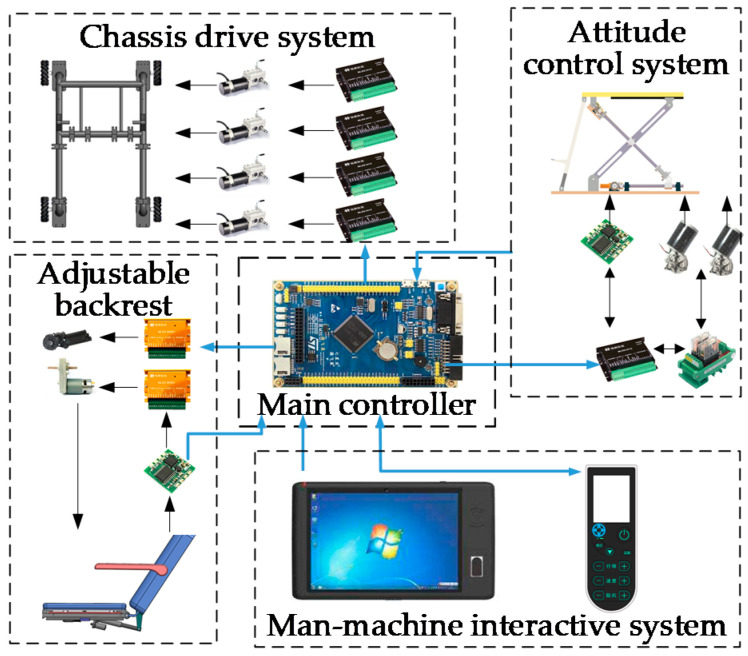
Intelligent toilet wheelchair control system.

Based on the above analysis, an intelligent toilet wheelchair control system was built to meet the toilet and walking requirements of disabled patients, as shown in Figure 15.

**Figure 16:**
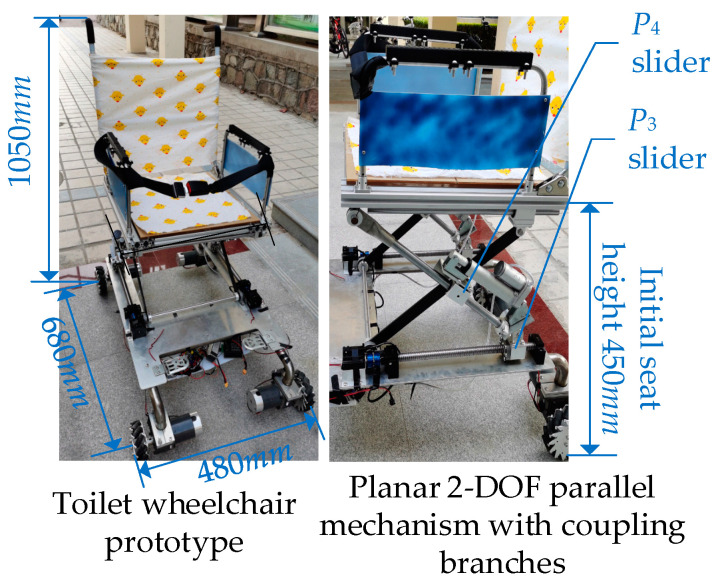
Principle prototype of the intelligent toilet wheelchair.

According to the corresponding functions, the control system was divided into five parts: a main control unit, a chassis walking system, an attitude adjustment system, a back-adjustment unit and a human–computer interaction system. Here, the main control unit is responsible for the management and operation of the whole wheelchair control system. The chassis walking unit consists of four Mecanum wheels and their driving motors to realize the omni-directional movement of the wheelchair. A gyroscope is arranged in both the attitude adjusting unit and the back adjusting unit to provide wheelchair attitude feedback and realize closed-loop control. The human–computer interaction unit is mainly used for the selection of the working mode, function parameter settings, operation status query and real-time display. Finally, the prototype design was completed, as shown in Figure 16.

### 5.2. Verification Experiment for Altitude and Attitude Adjustment

**Figure 17:**
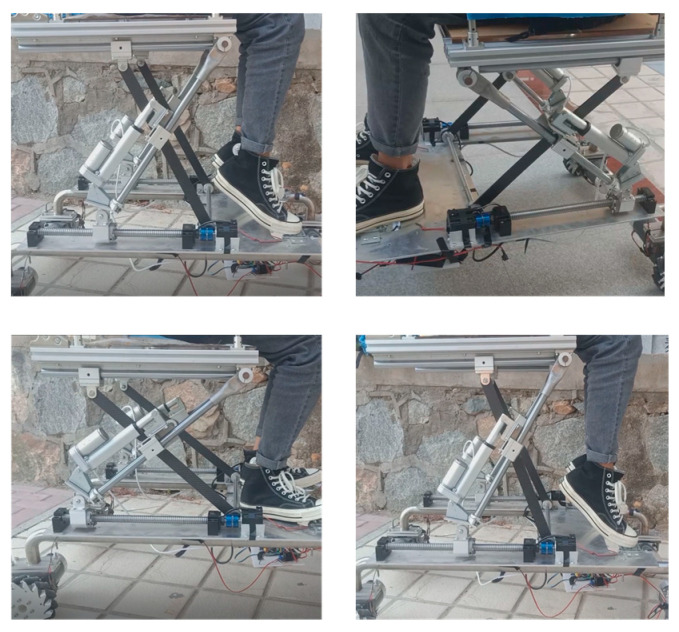
Experimental scenario.

**Figure 18:**
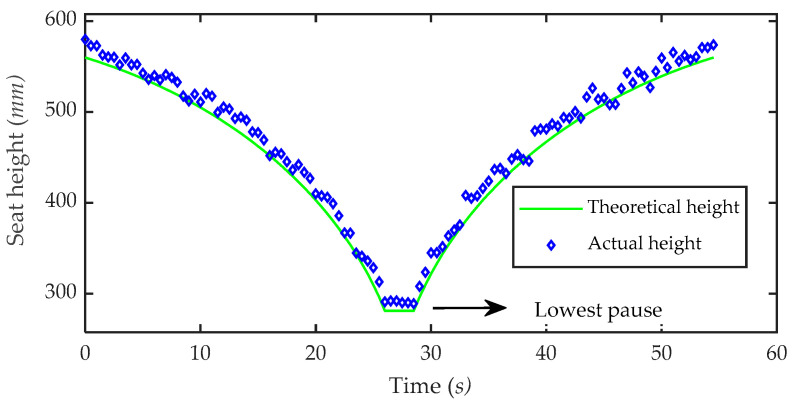
Experimental results of height adjustment.

In order to verify the adaptability of the toilet wheelchair to different toilet heights, a seat height adjustment experiment was carried out. Volunteers were invited to ride in the toilet wheelchair. The height of the wheelchair seat was adjusted to the highest position as the initial state. The ball screw used to adjust the height was set to move at a linear speed of 5 mm/s. In the process of the experiment, the seat moved downward from the highest to the lowest position, paused for a while and then moved to the highest position. During the whole movement, the seat was kept in a horizontal position, and measurements were taken every 0.5 s to record the height of the seat surface. The experimental process is shown in Figure 17, and the experimental results are shown in Figure 18.

From the experimental results, it can be seen that the actual height of the seat is close to the theoretical height curve, which shows the correctness of the seat height adjustment method. At the same time, it can be seen that the height adjustment range of the seat is 290~550 mm, which fully meets the requirements for different specifications of toilets. In addition, through observation, it can be found that the actual height of the seat is almost above the theoretical calculation height, and the deviation is consistent with the actual height. This is because the height of the seat from the ground includes the height of the parallel adjustment structure and the height of the chassis. The height of the chassis is constant, but due to processing and assembly errors, the actual value of this constant is larger than the theoretical value, resulting in the actual chair height exceeding the theoretical height. However, this deviation is not significant and does not affect the actual use effects. At the same time, this deviation can be controlled via the improvement of subsequent processing and the assembly process.

**Figure 19:**
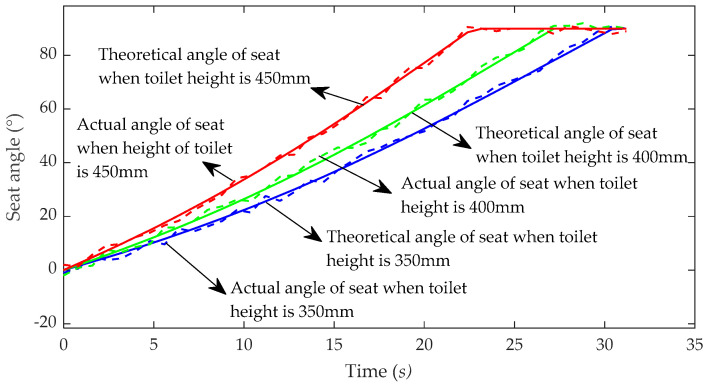
Experimental results of sit/stand attitude adjustment.

A posture adjustment experiment was carried out to verify that the toilet wheelchair can change the sitting/standing posture under different chair heights. As the patient is sitting on the chair surface, the adjustment of the patient’s posture can be expressed by the change in the tilt angle of the chair surface. The height of the toilet was mainly between 380 and 430 mm. The volunteers were asked to adjust the sitting/standing posture for chair heights of 350 mm, 400 mm and 450 mm. The angle-adjustable electric push rod moved at a speed of 5 mm/s, and measurements were taken every 0.8 s to record the inclination angle of the chair seat. The experimental results are shown in Figure 19.

It can be seen from the figure that the seat inclination can be adjusted from 0° to 90° at different heights. The inclination of 0° indicates that the chair seat is in the horizontal state, which represents the patient’s sitting posture, and the inclination of 90° indicates that the chair seat is in the vertical state, which represents the patient’s standing posture. Therefore, the toilet wheelchair meets the needs of patients for adjusting the sitting/standing posture at different heights. In addition, it can be seen that the posture adjustment capability of the toilet wheelchair is different at different heights, and the posture adjustment can be completed faster when the chair seat is higher than the ground. This is because the initial values of *θ*_1_ and *θ*_2_ are larger when the seat is higher, and they are more sensitive to the change in the length of the moving link. At the same time, it can also be seen that after the inclination of the chair seat reaches 90°—that is, after the patient completes the standing posture—the inclination of the chair seat does not continue to increase. This is because the inclination of the chair seat is limited, which can avoid excessive inclination and effectively protect the patient’s safety.

## 6. Summary

In this paper, the design and analysis of a toilet wheelchair for disabled people were studied. Through the analysis of human body size and the toilet use process, a design scheme for a planar two-degree-of-freedom parallel mechanism with coupling branch chains was proposed. A parallel mechanism is used as the posture adjustment mechanism of the left and right sides of the toilet wheelchair, which can reserve enough space for the sitting toilet under the seat so that the toilet wheelchair can be placed on the sitting toilet. By first decoupling the model and then coupling it, the degree of freedom of the parallel mechanism with coupling branch chains was calculated, and it was determined that the mechanism can meet the requirements for wheelchair height and angle adjustment at the same time. The kinematics, dynamics, workspace and singularity analyses laid the foundation for the realization of attitude adjustment. Through analysis of the constraints in the process of using a toilet, the actual size of the connecting rod of the parallel mechanism was determined, and the conversion from the theoretical model to the physical model was completed. Next, the coupling relationship between seat height and seat inclination adjustment ability was analyzed, and the seat height adjustment strategy under different road conditions was discussed. Then, the trajectory planning of attitude adjustment was carried out, and the weighted average function was used to find the optimal fixed point of the horizontal moving slider so that the actual trajectory of the end is close to the ideal trajectory under the single driving mode. Finally, the feasibility of the overall design scheme was verified by the height and angle adjustment experiments.

## 7. Conclusions

(1) The toilet wheelchair design scheme proposed in this paper adopts a planar 2-DOF parallel mechanism with coupling branch chains, which can adjust the height and the sitting/standing posture in order to facilitate the patient’s independent toilet use.

(2) The height adjustment range of the toilet wheelchair is 290~550 mm, and the tilt angle adjustment range is 0~90°, thus adapting to different specifications of toilets.

(3) This study only carried out preliminary experimental verification for the toilet use process. Follow-up work will further verify the adaptability to complex road conditions in the process of driving. In addition, the structural design and processing technology of the toilet wheelchair itself should be optimized.

## Figures and Tables

**Table 3:** Boundary conditions of evaluation indexes.

Evaluation Index	Boundary Conditions
Maximum reachable angle *θ*_max_	$L_{1}=\xi /5$ and $L_{2}=7\xi /8$
Minimum reachable angle *θ*_min_	$L_{1}=\xi /5$ and $L_{2}=2\xi /5$
Maximum reachable height *H*_max_	Either $L_{1}=\xi /5$ and $L_{2}=L_{6}/2$ or $L_{1}=\sqrt{\left|L_{6}^{2}-L_{8}^{2}\right|}/2$ and $L_{2}=L_{6}/2$
Minimum reachable height *H*_min_	Either $L_{1}=5\xi /12$ and $L_{2}=L_{6}/2$ or $L_{1}=\xi /5$ and $L_{2}=L_{6}/2$

**Table 4:** The maximum load on the nut base during attitude adjustment.

Horizontal Load (N)	Vertical Load (N)	L1 Length under Maximum Load (mm)	L2 Length under Maximum Load (mm)
412.5	250	280	220

**Table 5:** Equivalent elastic strain and total deformation data of lead screw.

Support Mode	Equivalent Elastic Strain	Total Deformation (mm)
Fixed at both ends	2.0156 × 10^−4^	2.1164 × 10^−2^
One end fixed, one end free	2.0156 × 10^−4^	2.7707 × 10^−2^

## Data Availability

Not applicable.
